# Chenodeoxycholic acid rescues axonal degeneration in induced pluripotent stem cell-derived neurons from spastic paraplegia type 5 and cerebrotendinous xanthomatosis patients

**DOI:** 10.1186/s13023-023-02666-w

**Published:** 2023-04-06

**Authors:** Yongchao Mou, Ghata Nandi, Sukhada Mukte, Eric Chai, Zhenyu Chen, Jorgen E. Nielsen, Troels T. Nielsen, Chiara Criscuolo, Craig Blackstone, Matthew J. Fraidakis, Xue-Jun Li

**Affiliations:** 1 Department of Biomedical Sciences, University of Illinois College of Medicine Rockford, Rockford, IL 61107 USA; 2 Department of Bioengineering, University of Illinois at Chicago, Chicago, IL 60607 USA; 3 Neurogenetics Clinic & Research Laboratory, Rigshospitalet, University of Copenhagen, Copenhagen, Denmark; 4 Department of Neuroscience, Reproductive Sciences and Odontostomatology, Federico II University, Naples, Italy; 5 Movement Disorders Division, Department of Neurology, Massachusetts General Hospital, Boston, MA 02114 USA; 6 MassGeneral Institute for Neurodegenerative Disease, Massachusetts General Hospital, Charlestown, Boston, MA 02129 USA; 7 Rare Neurological Diseases Unit, Department of Neurology, Attikon University Hospital, Medical School of the University of Athens, Athens, Greece

**Keywords:** Cerebrotendinous xanthomatosis, Spastic paraplegia type 5, Axonal degeneration, Chenodeoxycholic acid, Induced pluripotent stem cell

## Abstract

**Background:**

Biallelic mutations in *CYP27A1* and *CYP7B1*, two critical genes regulating cholesterol and bile acid metabolism, cause cerebrotendinous xanthomatosis (CTX) and hereditary spastic paraplegia type 5 (SPG5), respectively. These rare diseases are characterized by progressive degeneration of corticospinal motor neuron axons, yet the underlying pathogenic mechanisms and strategies to mitigate axonal degeneration remain elusive.

**Methods:**

To generate induced pluripotent stem cell (iPSC)-based models for CTX and SPG5, we reprogrammed patient skin fibroblasts into iPSCs by transducing fibroblast cells with episomal vectors containing pluripotency factors. These patient-specific iPSCs, as well as control iPSCs, were differentiated into cortical projection neurons (PNs) and examined for biochemical alterations and disease-related phenotypes.

**Results:**

CTX and SPG5 patient iPSC-derived cortical PNs recapitulated several disease-specific biochemical changes and axonal defects of both diseases. Notably, the bile acid chenodeoxycholic acid (CDCA) effectively mitigated the biochemical alterations and rescued axonal degeneration in patient iPSC-derived neurons. To further examine underlying disease mechanisms, we developed CYP7B1 knockout human embryonic stem cell (hESC) lines using CRISPR-cas9-mediated gene editing and, following differentiation, examined hESC-derived cortical PNs. Knockout of CYP7B1 resulted in similar axonal vesiculation and degeneration in human cortical PN axons, confirming a cause-effect relationship between gene deficiency and axonal degeneration. Interestingly, CYP7B1 deficiency led to impaired neurofilament expression and organization as well as axonal degeneration, which could be rescued with CDCA, establishing a new disease mechanism and therapeutic target to mitigate axonal degeneration.

**Conclusions:**

Our data demonstrate disease-specific lipid disturbances and axonopathy mechanisms in human pluripotent stem cell-based neuronal models of CTX and SPG5 and identify CDCA, an established treatment of CTX, as a potential pharmacotherapy for SPG5. We propose this novel treatment strategy to rescue axonal degeneration in SPG5, a currently incurable condition.

**Supplementary Information:**

The online version contains supplementary material available at 10.1186/s13023-023-02666-w.

## Background

Cholesterol and its bile acid derivatives play critical roles in maintaining the proper structure and function of the central nervous system (CNS) [1, 2]. Dysregulated cholesterol and bile acid homeostasis contributes to various neurological diseases, including motor neuron degenerative diseases [3, 4]. There are two main pathways in cholesterol degradation. First, the ‘classic’ pathway is initiated by 7α-hydroxylation of cholesterol through cytochrome P450 family 7 subfamily A member 1 (CYP7A1). Second, in the ‘acidic’ pathway that predominates in the CNS, cholesterol is alternatively oxidized at side chains, producing the oxysterols 25-hydroxycholesterol (25-OHC) and 27-hydroxycholesterol (27-OHC) that are α-hydroxylated by cytochrome P450 Family 7 Subfamily B Member 1 (CYP7B1) [5]. Defects in cholesterol and bile acid degradation are associated with severe progressive neurodegenerative diseases such as hereditary spastic paraplegia type 5 (SPG5) and cerebrotendinous xanthomatosis (CTX).

Hereditary spastic paraplegias (HSPs) are a heterogeneous group of neurologic disorders characterized by progressive spasticity and weakness of the lower limbs due to length-dependent dysfunction of corticospinal motor neurons [6, 7]. Over 80 different genetic loci (SPG1-87, plus others) have been linked to HSPs [8]. SPG5 is a rare, autosomal recessive HSP caused by loss-of-function of CYP7B1, which catalyzes the degradation of cholesterol into primary bile acids through the ‘acidic’ pathway [5]. *CYP7B1* mutations lead to significant accumulation of both 27-OHC and 25-OHC [5, 9], which are toxic to neurons and contribute to motor neuron degeneration by inducing aberrant neuronal morphology and impairing synaptic function [10–12].

Another critical enzyme in cholesterol degradation is cytochrome P450 oxidase, also known as sterol 27-hydroxylase, encoded by *CYP27A1*. Biallelic *CYP27A1* mutations cause CTX, a rare and autosomal recessive bile acid biosynthesis and sterol storage disorder [13]. CTX is a neurometabolic, multisystem disorder clinically characterized by the tell-tale triad of juvenile cataract, progressive ataxia, and tendon xanthomas, plus chronic diarrhea and various childhood-onset progressive neuropsychiatric symptoms [14]. The inborn defect of sterol 27-hydroxylase disturbs cholesterol degradation, impairs the production of the bile acids cholic and chenodeoxycholic (CDCA), and results in the overproduction of cholesterol, cholestanol (5α-dihydrocholesterol) and other bile alcohols. These metabolites accumulate in various tissues where cholestanol deposition far exceeds that of other sterols, hence the alternative description cholestanolosis for CTX [15, 16]. Early institution of oral treatment with CDCA is vital to halt disease progression and improve prognosis.

A characteristic pathologic change in both CTX and SPG5 is degeneration of corticospinal motor neuron axons [17]. However, how axons of these cortical PNs degenerate, and whether axonal defects of human cortical neurons can be effectively mitigated by targeting cholesterol and bile acid metabolism, remain unknown. Induced pluripotent stem cells (iPSCs) [18–20] capable of differentiating into various neuronal subtypes provide unique sources and tools to generate disease-specific neurons for investigating cellular and subcellular pathological mechanisms as well as to test potential therapeutic drugs. In this study, we generated CTX and SPG5 iPSC lines from patient skin fibroblasts and then differentiated them into cortical PNs to establish human neuronal models for CTX and SPG5. Using these iPSC-based models as a paradigm for disease-specific biochemical and axonal phenotypes, we examined the role of impaired cholesterol and bile acid metabolism in the pathogenesis of SPG5 and CTX, with a goal of identifying potential rational treatment approaches.

## Methods

### Reprogramming fibroblasts of CTX and SPG5 patients into iPSCs

Skin biopsies were collected from CTX and SPG5 patients, and fibroblast cell lines were generated and maintained using standard methods. The CTX patient has compound heterozygous missense mutations of c.397T > C and c.1183C > T in the *CYP27A1* gene, leading to amino acid changes p.Trp133Arg and p.Arg395Cys, respectively. The SPG5 patient has compound heterozygous mutations in the *CYP7B1* gene, comprising a premature stop codon (c.334C > T, p.Arg112*) and an amino acid change (c.1456C > T, p.Arg486Cys) [21]. Of the three missense mutations, two (c.1183C > T and c.1456C > T) have been reported to be pathogenic or likely pathogenic (NCBI ClinVar). The SPG5 patient exhibited characteristic lower limb spasticity and weakness (patient 1 in Ref. [21]). The CTX patient, a 32-year-old man with disease onset at two years old, had chronic diarrhea, bilateral cataracts, mental retardation, ataxia, and spastic paraplegia. Informed consent was obtained for collecting skin biopsies. The study procedure was approved by the Ethics Committee of the Capital Region of Denmark.

To establish iPSC lines using episomal transduction, ~ 200,000 fibroblasts were dissociated and transfected with episomal vectors (Addgene) containing the pluripotency factors Oct3/4, Sox2, L-Myc, and Klf4, as previously reported [22]. After electroporation transduction, cells were plated and cultured in DMEM supplemented with 10% fetal bovine serum. After one week in culture, cells were then dissociated, plated onto irradiated mouse embryonic fibroblast (MEF) feeders, and cultured in human embryonic stem cell (hESC) medium supplemented with fibroblast growth factor (FGF)-2 (10 ng/mL, PeproTech, Cat. #: 100-18B). hESC medium contains DMEM/F12 (Corning, Cat. #: 10-092-CV), 1 × non-essential amino acids (NEAA, Gibco, Cat. #: 11140-050), 20% Knockout Serum Replacement (Gibco, Cat. #: A31815), 0.5 × GlutaMax (Gibco, Cat. #: 35050-061), and 0.1 mM β-mercaptoethanol (Sigma-Aldrich, Cat. #: M3148). Between days 20 ~ 30, iPSC-like clones were selected, followed by expansion and detailed characterization. The maintenance of disease-specific mutations after reprogramming was confirmed in patient iPSCs. Human iPSC clones derived from normal individuals were generated previously [23] and used as controls (wild-type, WT).

### Cortical PNs differentiation from human iPSCs

Cortical PNs were differentiated from human iPSCs using our previously published methods [24, 25]. Briefly, human iPSCs were detached from feeder cells and cultured in suspension for 4 days. The resulting stem cell aggregates were then transferred to neural induction medium (NIM) supplemented with 2 µM DMH1 and 2 µM SB431542 for 3 additional days in suspension; NIM was prepared by supplementing DMEM/F12 medium with 1 × N2 (Gemini Bio-Products, Cat. #: 400-163), 2 µg/ml heparin (Sigma-Aldrich, Cat. #: H3149), and 1 × NEAA (Gibco, Cat. #: 11140-050). Cell aggregates were then attached onto 6-well plates and cultured in NIM to induce cells to form neuroepithelial (NE) cells. At day 17, NE cells were mechanically isolated and then cultured in suspension in NIM supplemented with 1 × B27, 1 μM cAMP, and 10 ng/mL IGF-1 to generate neurospheres. After day 42, neurospheres were plated onto poly-ornithine- and Geltrex-coated coverslips at a density of about 20,000 cells per coverslip. Cortical PNs were cultured in neural differentiation medium (NDM) containing Neurobasal medium, 1 × N2, 1 μM cAMP, 10 ng/mL IGF-1, 10 ng/mL BDNF and 10 ng/mL GDNF.

### Immunofluorescence staining

To characterize iPSCs and cortical PNs, immunofluorescence staining was performed as described previously [26]. Cells were fixed with cold 4% paraformaldehyde for 20 min, permeabilized with 0.2% Triton X-100 for 10 min, and blocked with 10% donkey serum for one hour. Fixed cells were then incubated with primary antibodies at 4 °C overnight, followed by fluorescence-conjugated secondary antibodies. Primary antibodies used in this study included mouse monoclonal IgM anti-Tra-1-60 (Santa Cruz Biotechnology, Cat. #: Sc-21705, 1:50), mouse monoclonal IgG anti-SSEA-4 (DSHB, Cat. #: MC-813-70, 1:100), goat polyclonal IgG anti-NANOG (R&D Systems, Cat. #: AF1997, 1:500), rat monoclonal IgG anti-CTIP2 (Abcam, Cat. #: Ab18465, 1:2000), rabbit polyclonal IgG anti-TAU (Sigma-Aldrich, Cat. #: T6402, 1:100), and mouse IgG anti-phosphorylated neurofilament-heavy-chain (pNfH) (Millipore, Cat. #: MAB1592, 1:1,000). Nuclei were stained with Hoechst. Fluorescence immunostaining was visualized using an Olympus IX83 microscope and an Olympus confocal microscope.

### Axonal length and swellings

For examining axonal outgrowth, cortical PNs were stained with TAU (axonal marker) and CTIP2 (cortical projection neuron marker) after being dissociated and grown on coverslips for 48 h. Axonal length was measured using the NeuronJ plugin for Fiji software [27, 28]; the longest process with the greatest tau intensity from CTIP2^+^ neurons was measured. A minimum of 50 cells were measured in blindly-selected fields from 3 independent coverslips, as previously described [26, 28]. Axonal swellings were measured by immunostaining for TAU after long-term culture (about 3 months). At least 3 random fields each coverslip were selected and imaged from three independent coverslips by a person who was blinded to the experimental groups. The number of axonal swellings was counted and divided by the total axonal length in each field, measured using Fiji software. Axonal swellings were identified as those structures with a diameter > 2 times that of the diameter of the contiguous axon [29, 30].

### Cholesterol quantification

Cholesterol content in cortical PNs was measured using a cell-based cholesterol detection assay kit (Cat. #:10009779, Cayman Chemical) and Total Cholesterol Assay kit (Colorimetric, Cat. #: STA-384, Cell Biolabs) per the manufacturers’ instructions. Filipin staining was visualized using an Olympus IX83 microscope under the same exposure times. At least five images were randomly taken from each of three different coverslips. Average Filipin intensities were quantified using Fiji software. Briefly, average Filipin intensities in neuronal cell bodies and axons were traced using the “segmented line” tool in Fiji software, and the pixel intensity of Filipin in neuronal cell bodies and axons was obtained using the “profile plot” function in Fiji. Axons were identified based on morphological criteria (constant thin diameter, long neurites with no branching, and direct emergence from the cell body), as described previously [26]. Total cholesterol content of WT and CTX cortical PNs was determined per the manufacturer’s instructions. The concentration of cholesterol per million cells was calculated by comparison to the cholesterol standard curve.

### *CYP7B1* knockout hESC lines

To knockout (KO) *CYP7B1* in hESCs, CYP7B1 CRISPR-cas9 KO plasmids containing RNA sequences that specifically target the *CYP7B1* gene were obtained from Santa Cruz Biotechnology (Cat. #: sc-405345). KO and homology-directed repair plasmids (also expressing cas9 and the drug selection gene puromycin) were electroporated into hESCs following the manufacturer’s protocol. After electroporation, cells were plated on MEF feeder cells in hESC medium with bFGF. After drug selection, hESC clones with *CYP7B1* KO were selected, expanded, and differentiated into cortical PNs for characterization and phenotypic analyses.

To examine the protein levels of CYP7B1, sandwich ELISA analysis was performed in cell lysates using the Immunotag human CYP7B1 ELISA kit (GBioscience, Cat. #: IT4723) following the manufacture’s protocol. The concentration of CYP7B1 was calculated using the CYP7B1 standard curve. Expression levels of CYP7B1 were compared between CYP7B1 knockout cells and H9 normal control cells.

### Real-time quantitative PCR

Total RNA was isolated from fibroblasts, stem cells, neuroepithelial cells, and neurons using TRIzol. One microgram of RNA was used to generate cDNA using the High-Capacity cDNA Reverse Transcription Kit. Real-time PCR was performed using the PowerUp SYBR Green Master Mix in the QuantStudio 6 Flex Real-Time PCR System. PCR cycling conditions were: 50 °C for 2 min, 95 °C for 3 min, 45 two-step cycles at 95 °C for 15 s and 60 °C for 60 s, followed by a melt-curve stage at 95 °C for 15 s, 60 °C for 60 s, and 95 °C for 15 s. Primers used in this study include OCT4, 5′-TATGCAAAGCAGAAACCCTCGTGC-3′, 5′-TTCGGGCACTGCAGGAACAAATTC-3′; FGF5, 5′-AGCAGTAGCGCTATGTCTTCCTCT-3′, 5′-AAACTGCTCTGCTCCAAGCCACTT-3′; PAX6, 5′-TCTTTGCTTGGGAAATCCG-3′, 5′-CTGCCCGTTCAACATCCTTAG-3′; NFL, 5′-ATGAGTTCCTTCAGCTACGAGC-3′, 5′-CTGGGCATCAACGATCCAGA-3′; CYP7B1, 5′-GCTGCAGTCAACAGGTCAAA-3′, 5′-CAGTAGTCCCCGGTCTCTGA-3′; and TAU, 5′-GACAGAGTCCAGTCGAAGATTG-3′, 5′-AGGTCAGCTTGTGGGTTTC-3′.

### Statistical analysis

Statistical significance of mean values among multiple groups was analyzed with Tukey's range test after ANOVA. Dunnett’s test was used to compare multiple groups with one group. Two-sided *t*-tests were used to examine the statistical significance between two groups. The significance level was defined as *p* < 0.05.

## Results

### Generation and characterization of CTX iPSCs

**Fig. 1:**
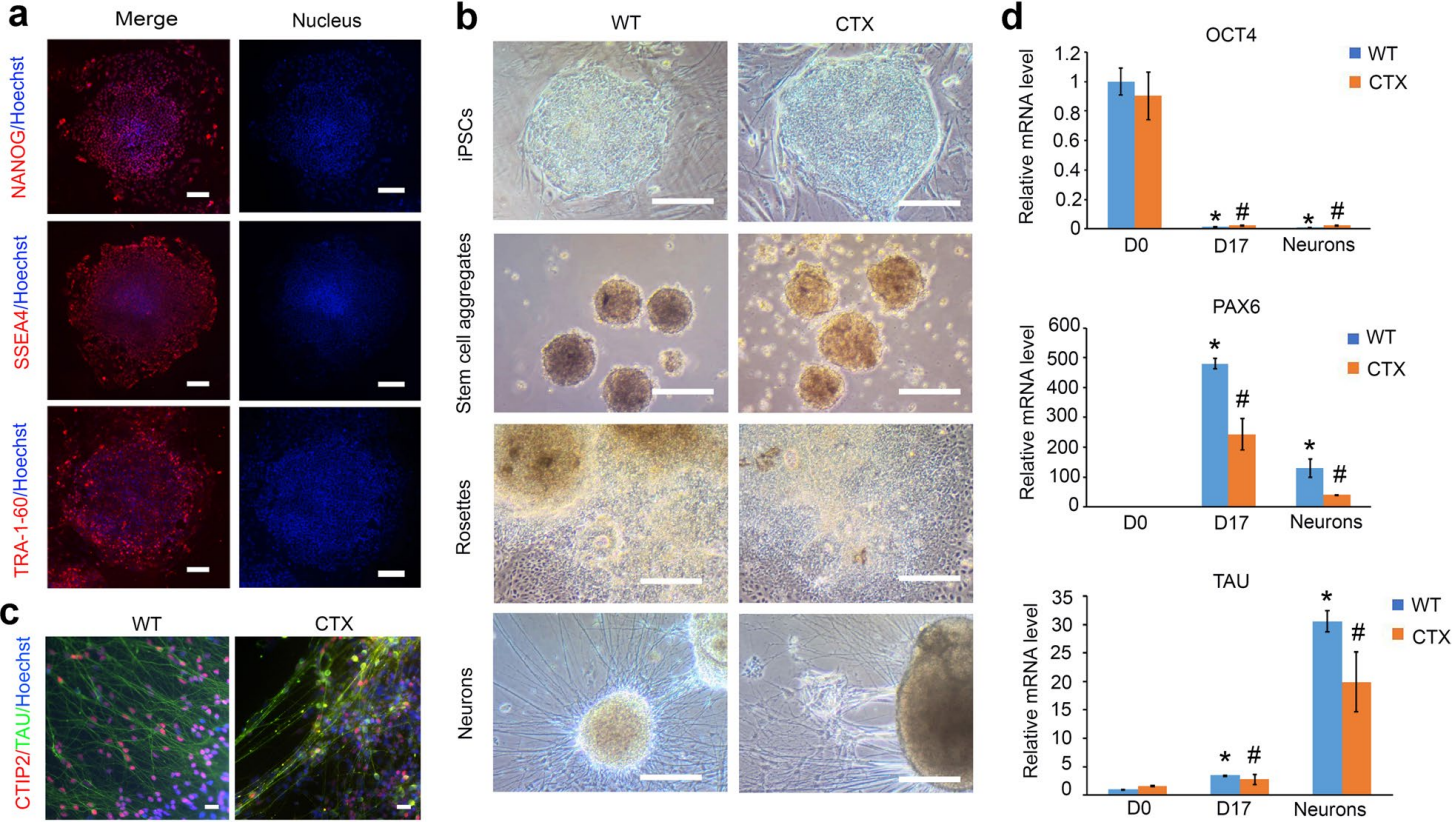
CTX iPSC characterization and cortical PN differentiation. **a** Immunostaining of NANOG, SSEA4, and TRA-1-60 in CTX iPSCs. Red: NANOG, SSEA, TRA-1-60, respectively. Blue indicates Hoechst-stained nuclei. Scale bar, 100 µm. **b** Representative phase-contrast images of CTX iPSCs at different stages of cortical PN differentiation including stem cell colony, stem cell aggregates (also known as embryoid bodies), neuroepithelial cells (rosettes), and neurons. Scale bar: 200 μm. **c** Immunostaining images of CTIP2^+^ and TAU^+^ cortical PNs in WT and CTX neural cultures. Red: CTIP2; green: TAU; blue: Hoechst. Scale bar, 20 μm. **d** qPCR quantification of pluripotency (*OCT4*), neural progenitor (*PAX6*), and neuronal (*TAU*) gene expression at different stages of cortical PN differentiation in WT and CTX cells. Data are represented as means ± SEM. *p < 0.05 compared to D0 WT, ^#^*p* < 0.05 compared to D0 CTX by Dunnett’s test after ANOVA

In cholesterol metabolism, one critical gene for cholesterol oxidation is *CYP27A1*, which generates 27-hydroxycholesterol and is mutated in CTX [13]. To model this disease, CTX iPSCs were generated from dermal fibroblasts of a CTX patient with mutations in the *CYP27A1* gene using an integration-free episomal method by transducing cells with episomal vectors containing Oct3/4, Sox2, Klf4, and L-Myc [22]. CTX iPSC clones exhibited characteristic embryonic stem cell (ESC) morphology and expression of the pluripotency markers NANOG, SSEA4, and TRA-1-60 by immunostaining (Fig. 1a). CTX and WT iPSCs were then differentiated into cortical PNs using our well-established protocol [24, 25].

CTX iPSCs were successfully differentiated into forebrain glutamatergic neurons through the same stages as WT cells, including stem cell aggregates, neuroepithelial cells with rosettes, neural progenitors in suspension, and cortical glutamatergic neurons with projected neurites (Fig. 1b). These cortical PNs were positive for the subcerebral projection neuron marker CTIP2 and the axonal marker TAU, indicating successful differentiation of cortical PNs from CTX iPSCs (Fig. 1c). Furthermore, the expression of different stage markers was determined by qRT-PCR, including the pluripotency marker (*OCT4*), neural stem marker (*PAX6*), and neural marker (*TAU*). The mRNA expression of *OCT4* was significantly reduced during differentiation. The expression of *PAX6* peaked in D17 neural progenitor cells and then declined with further differentiation. In parallel, *TAU* expression was gradually increased and had the highest expression during the later neural stage (Fig. 1d). *OCT4* and *TAU* expression did not show significant differences at the same stages of differentiation towards cortical PNs between CTX and WT groups (Fig. 1d). These data indicate that we successfully generated human CTX iPSC lines and that the *CYP27A1* mutation did not affect the differentiation of CTX iPSCs along the forebrain glutamatergic neuron lineage.

**Fig. 2:**
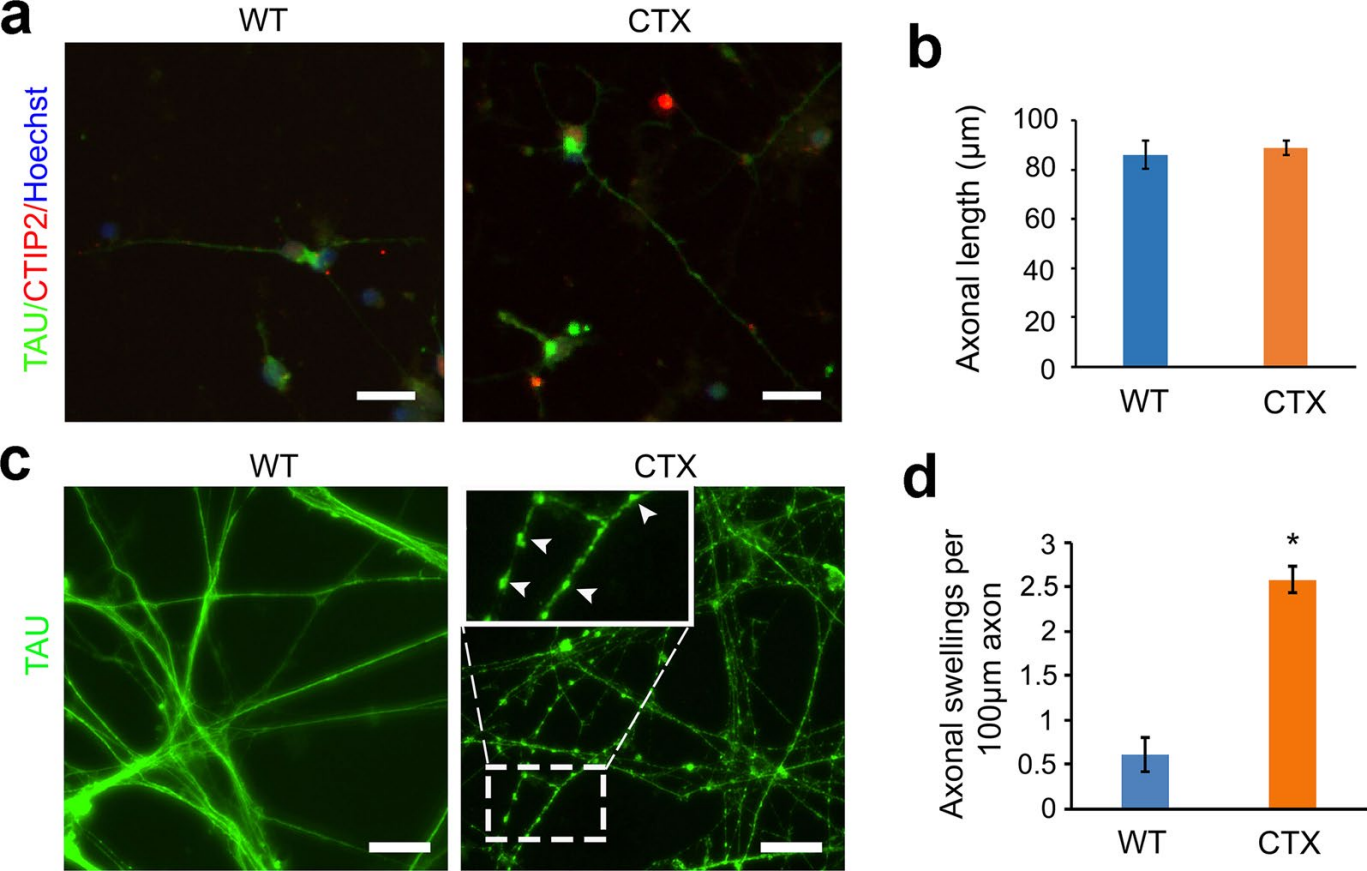
Axonal degeneration of cortical PNs derived from CTX iPSCs. **a** Representative images showing neurite outgrowth of WT and CTX cortical PNs, 2 days after plating, with immunostaining for CTIP2 and TAU. Red: CTIP2; green: TAU; blue: Hoechst. Scale bar, 20 μm. **b** Quantification of axonal outgrowth of WT and CTX cortical PNs. Data are represented as means ± SEM, with no significant differences between CTX and WT groups. **c** Representative pictures of axonal swellings, with immunostaining for TAU in WT and CTX cortical PNs. Accumulated axonal swellings were observed in CTX neurons; representative images of axonal swellings are magnified and indicated with arrowheads. Green: TAU. Scale bar: 20 μm. **d** Quantitative graph showing axonal swellings of WT and CTX cortical PNs. Data are represented as means ± SEM. *p < 0.05 compared to WT by two-sided Student *t*-test

Neurodegeneration, especially cortical PN degeneration leading to motor impairment, is a characteristic pathology in CTX patients [31, 32]. We examined axonal outgrowth and swellings of human cortical PNs derived from CTX patient iPSCs (Fig. 2a–d). D42 CTX and WT control neurons were plated onto coverslips after dissociation and stained for CTIP2 and TAU to visualize cortical PNs and axons (Fig. 2a). At 2 days after plating, axonal length of CTX cortical PNs did not show significant changes compared with WT neurons (Fig. 2b). In long-term cultures, the axonal length of some CTX forebrain neurons was dramatically reduced (data not shown), suggesting degeneration of cortical PN axons in CTX. To directly assess axonal degeneration in CTX neurons, we examined axonal swellings, a characteristic pathology observed in multiple forms of HSP [33–36], in long-term-cultured cortical PNs by immunostaining for TAU (Fig. 2c). The number of axonal swellings was significantly increased in CTX forebrain neurons as compared to WT neurons (Fig. 2d). Thus, CTX cortical PNs exhibit axonal impairments, as indicated by accumulation of axonal swellings in long-term cultures.

### Impaired cholesterol metabolism and axonal defects were rescued by CDCA in CTX cortical PNs

**Fig. 3:**
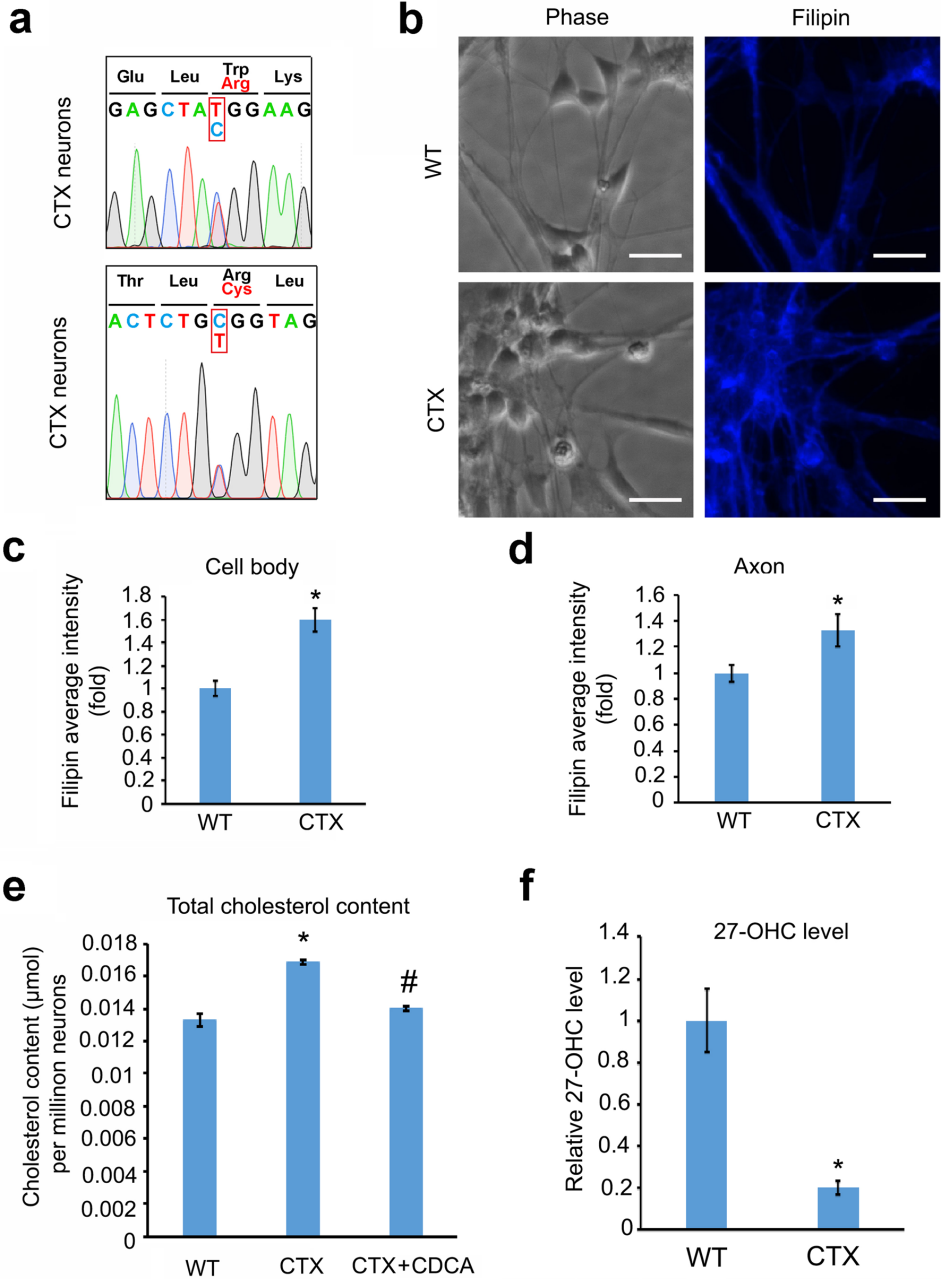
Cholesterol accumulation in CTX cortical PNs. **a** Genomic DNA sequencing confirmed the compound heterozygous mutations c.397 T > C, p.Trp133Arg and c.1183C > T, p.Arg395Cys in the *CYP27A1* gene in CTX PNs. **b** Representative images showing Filipin staining in WT and CTX cortical PNs. Blue: Filipin. Scale bar, 20 μm. **c** and **d** Quantitative graphs showing the Filipin intensity fold change in cell body (**c**) and axon (**d**) of WT and CTX cortical PNs. Filipin staining intensities in CTX neurons were significantly increased compared to control neurons. **e** Total cholesterol content in WT and CTX cortical PNs, and CTX cortical PNs treated with CDCA. **f** Relative 27-OH-cholesterol levels in CTX cortical PNs were significantly reduced compared to WT neurons. Data are represented as means ± SEM. *p < 0.05 compared to WT by two-sided Student *t*-test (for **c, d** and **f**). *p < 0.05 compared to WT, ^#^*p* < 0.05 compared to CTX control by Tukey’s range test after ANOVA (for **e**)

Biallelic, loss-of-function mutations in the *CYP27A1* gene result in abnormal cholesterol metabolism and pathological cholesterol accumulation in CTX patients [15]. In CTX iPSC-derived cortical PN cultures, genomic DNA sequencing revealed compound heterozygous mutations of c.397T > C (i.e., p.Trp133Arg) and c.1183C > T (i.e., p.Arg395Cys) in the *CYP27A1* gene (Fig. 3a). These missense mutations are the same as in the patient’s fibroblast cells, confirming persistence of CTX disease mutations after reprogramming. We examined whether CTX iPSCs-derived cortical PNs exhibit pathological cholesterol accumulation. Cholesterol content in CTX cortical PNs was determined by both Filipin staining and a colorimetric cholesterol assay (Fig. 3b–e). Quantifications of Filipin staining showed a significant increase of cholesterol in the cell body and axons of CTX neurons as compared with WT neurons (Fig. 3b–d). Cholesterol accumulation in CTX forebrain neurons was confirmed using the colorimetric cholesterol assay (Fig. 3e). Moreover, because of impaired oxidation of cholesterol, 27-OHC levels in CTX neurons were significantly reduced (Fig. 3f), confirming the recapitulation of disease-specific biochemical changes in patient iPSC-derived neurons.

Due to cholesterol metabolic impairment, the downstream product CDCA is deficient in CTX patients. CDCA also has a feedback effect on regulating cholesterol levels. Although CDCA has been used to decrease bile acid and cholesterol metabolic defects in clinical trials [37, 38], it remains largely unknown whether CDCA can mitigate axonal degeneration and reduce cholesterol accumulation in human neurons. Thus, we examined if CDCA can efficiently reduce cholesterol accumulation in CTX forebrain neurons (Fig. 3e). Total cholesterol colorimetric assays confirmed that CDCA treatment (10 µM for one week) dramatically reduced cholesterol content in CTX neurons (Fig. 3e), while the cholesterol content in normal neurons was not significantly affected by CDCA (Additional file 1: Fig. S1), indicating a specific effect of CDCA on CTX neurons. Cholesterol levels in CTX neurons were restored to a level similar to WT neurons by CDCA (Fig. 3e), revealing that CDCA can normalize cholesterol homeostasis.

**Fig. 4:**
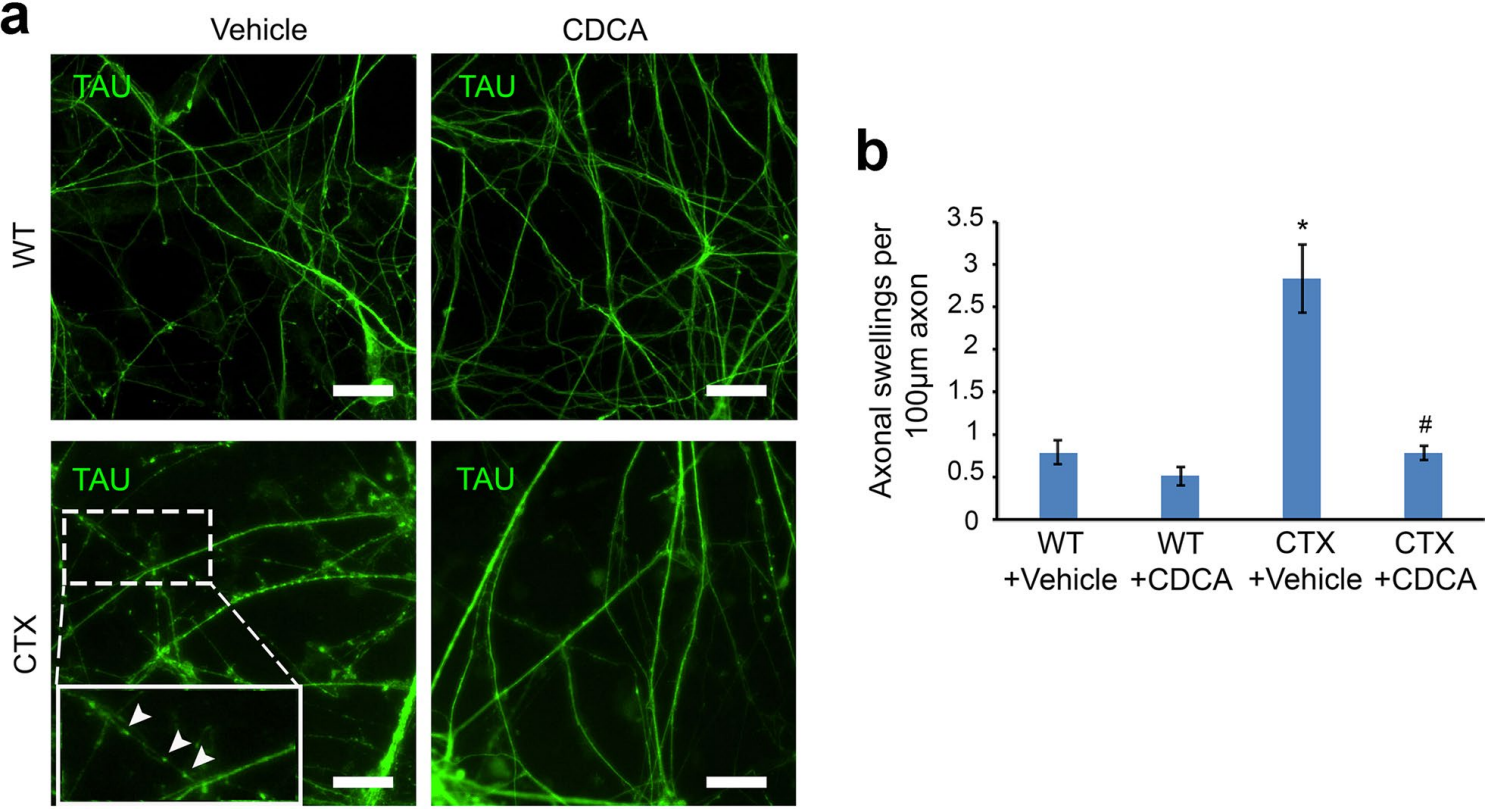
Axonal swellings of CTX cortical PNs after treatment with vehicle or CDCA. **a** Representative immunostaining images of TAU in WT and CTX cortical PNs after treatment with vehicle or CDCA. Accumulated axonal swellings are observed in CTX vehicle-treated neurons. Representative axonal swellings are magnified and indicated with arrowheads. Scale bar, 20 μm. **b** Quantitation of axonal swellings in WT and CTX cortical PNs after treatment with vehicle or CDCA. Axonal swellings in CTX neurons were significantly suppressed by CDCA treatment. Data are represented as means ± SEM. *p < 0.05 compared to WT groups, ^#^*p* < 0.05 compared to CTX + vehicle by Tukey’s range test after ANOVA

Given that accumulated axonal swellings are a characteristic pathology in CTX neurons, we next determined whether increased axonal swellings in these neurons can be similarly rescued by CDCA treatment. In long-term cultures, we observed a significant increase in axonal swellings compared with WT neurons (Fig. 4a and b), as we found before (Fig. 2c and d). These cells were treated with CDCA (10 µM) or vehicle control (DMSO) for 7 days. After CDCA treatment, there was a significant decrease in axonal swellings in CTX neurons, while CDCA did not show significant effects on WT control neurons (Fig. 4a and b). To further elucidate the role of cholesterol homeostasis on axonal defects, we treated wild-type neurons with cholesterol (Additional file 1: Fig. S2), which led to accumulated axonal swellings and axon breakdown. Taken together, our data indicate that CTX iPSCs-derived cortical PNs can be used as a platform for evaluating disease-specific phenotypes and that CDCA significantly rescued axonal defects of CTX cortical PNs by restoring cholesterol homeostasis.

### Generation and characterization of SPG5 iPSC lines

**Fig. 5:**
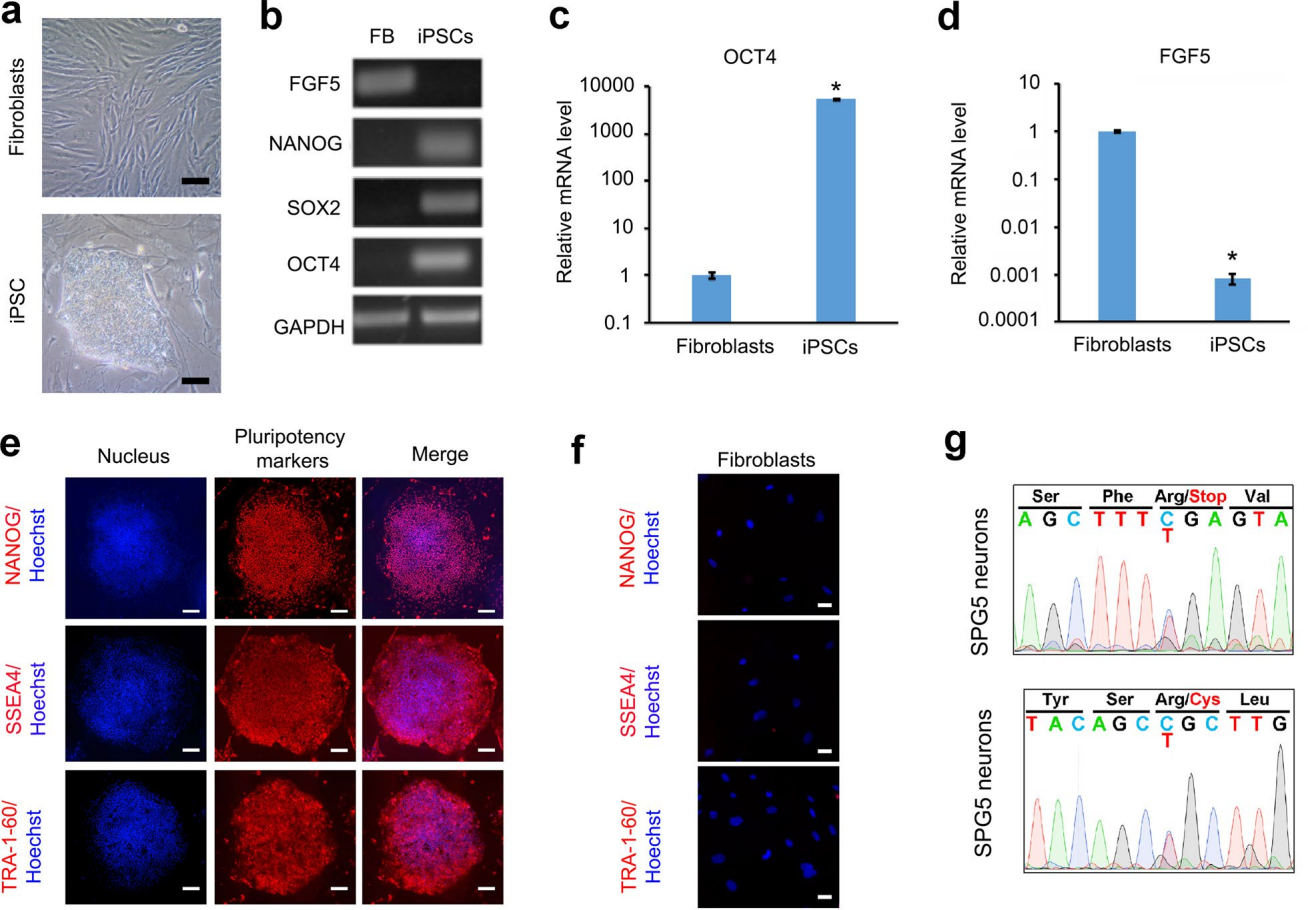
Establishment and characterization of SPG5 iPSCs. **a** Phase-contrast images of SPG5 dermal fibroblasts and iPSCs. **b** PCR gel images showing the expression of *FGF5*, *NANOG*, *SOX2*, and *OCT4* in SPG5 fibroblasts (FB) and iPSCs. GAPDH is a housekeeping gene. **c** and **d** The mRNA expression of *OCT4* and *FGF5* in fibroblasts and iPSCs by qRT-PCR. **e** Immunostaining showing the protein expression of pluripotency markers NANOG, SSEA4, and TRA-1-60 (red) in iPSCs. Blue: Hoechst. **f** No expression of pluripotency markers was detected in SPG5 fibroblasts. Blue: Hoechst. **g** Genomic DNA sequencing confirmed the compound heterozygous mutations c.334C > T, p.Arg112* and c.1456C > T, p.Arg486Cys in the *CYP7B1* gene. Data are represented as means ± SD. *p < 0.05 compared to fibroblast cells by two-sided Student’s *t*-test. Scale bars, 100 µm (**a**) and 50 µm (**e** and **f**)

SPG5 iPSCs were generated from dermal fibroblasts of a SPG5 patient with mutations in the *CYP7B1* gene using the same method as for CTX. After expansion, the SPG5 iPSCs showed typical ESC colony morphology (Fig. 5a). We further examined mRNA expression of pluripotency markers and fibroblast growth factor. *NANOG*, *OCT4*, and *SOX2* expression was observed in SPG5 iPSCs but not in fibroblasts, whereas *FGF5* was enriched in skin fibroblasts (Fig. 5b). Real-time PCR quantifications revealed that the pluripotency marker *OCT4* was highly expressed in iPSCs (Fig. 5c), while the fibroblast growth factor *FGF5* was highly expressed in fibroblasts and significantly reduced in iPSCs (Fig. 5d). As expected, immunostaining showed that iPSC clones uniformly expressed pluripotency proteins NANOG, SSEA4, and Tra-1-60 (Fig. 5e); no expression of these proteins was observed in fibroblasts (Fig. 5f). In SPG5 iPSC-derived neuron cultures, genomic DNA sequencing revealed compound heterozygous mutations of c.334C > T, p.Arg112* and c.1456C > T, p.Arg486Cys in the *CYP7B1* gene, confirming maintenance of disease mutations after reprogramming (Fig. 5g).

**Fig. 6:**
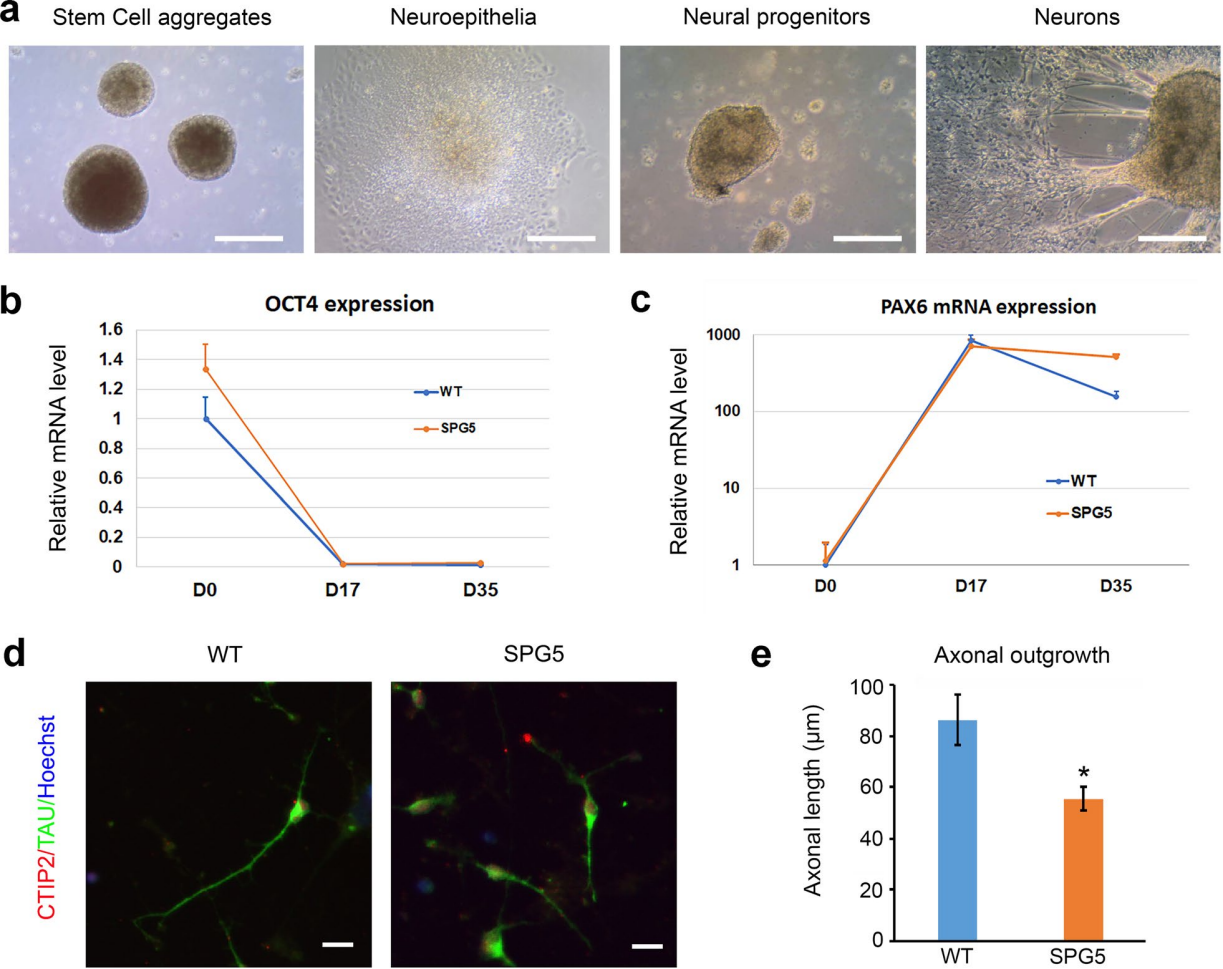
Reduced axonal outgrowth of SPG5 iPSC-derived cortical PNs. **a** Representative phase-contrast images showing different stages during differentiation of cortical PNs from SPG5 iPSCs. Scale bar, 100 µm. **b** and **c** qPCR quantification of *OCT4* and *PAX6* expression at different time points during the differentiation of cortical PNs from WT and SPG5 iPSCs: D0 (Day 0), stem cell stage. D17 (day 17), neuroepithelial cells, D35 (day 35), neurons. Expression of both genes at day 17 and day 35 was significantly altered compared to day 0 by Dunnett’s test. **d** Double immunostaining for TAU and CTIP2 in WT and SPG5 cortical PNs. Red: CTIP2; green: TAU; blue: Hoechst. Scale bar, 20 µm. **e** Axonal outgrowth quantification of WT and SPG5 cortical PNs revealed a significant reduction of axonal length in SPG5 cortical PNs compared to WT neurons. Data are represented as means ± SD. *p < 0.05 compared to WT by two-sided Student *t*-test

To examine the disease-related phenotypes, we differentiated SPG5 iPSC clones into cortical PNs, the cell type primarily affected in SPG5 patients, using our previous method. SPG5 iPSCs were successfully differentiated into cortical PNs, going through the same stages as WT cells (Fig. 6a). To evaluate neural differentiation, we examined expression of pluripotency (*OCT4*) and neural stem cell (*PAX6*) markers in SPG5 iPSCs during differentiation to cortical PNs (Fig. 6b and c). *OCT4* expression was significantly reduced in neuroepithelia (D17) and neurons (D35) compared with stem cells (D0), implying that pluripotency was decreased during the differentiation towards neurons; no significant differences were observed between WT and SPG5 cells (Fig. 6b). The mRNA expression of *PAX6* was significantly increased in neuroepithelia and neurons compared with stem cells (Fig. 6c). *PAX6* levels peaked at D17 and were comparable between WT and SPG5 neuroepithelia (Fig. 6c). Collectively, these data show that both markers appeared at their expected time in WT and SPG5 cells, suggesting that the SPG5 mutations in *CYP7B1* do not affect forebrain neural differentiation from iPSCs.

Axonal defects of cortical PNs are characteristic pathological phenotypes in HSPs, comprising impaired axonal outgrowth and accumulated axonal swellings [26, 28, 39]. To determine whether SPG5 iPSCs-derived cortical PNs recapitulate HSP-specific phenotypes, axonal outgrowth in WT and SPG5 cortical PNs was assessed by immunostaining (Fig. 6d, e). After dissociation with Accutase, WT and SPG5 cortical PNs were cultured onto coverslips for 48 h, followed by fixation and immunostaining of the axonal marker, TAU, and the subcerebral projection neuron marker, CTIP2 (Fig. 6d). CTIP2^+^ SPG5 cortical PNs had dramatically shorter axons compared with WT neurons (Fig. 6d, e). Thus, human SPG5 iPSCs-derived cortical PNs exhibited an impaired axonal outgrowth phenotype, with decreased axonal length.

**Fig. 7:**
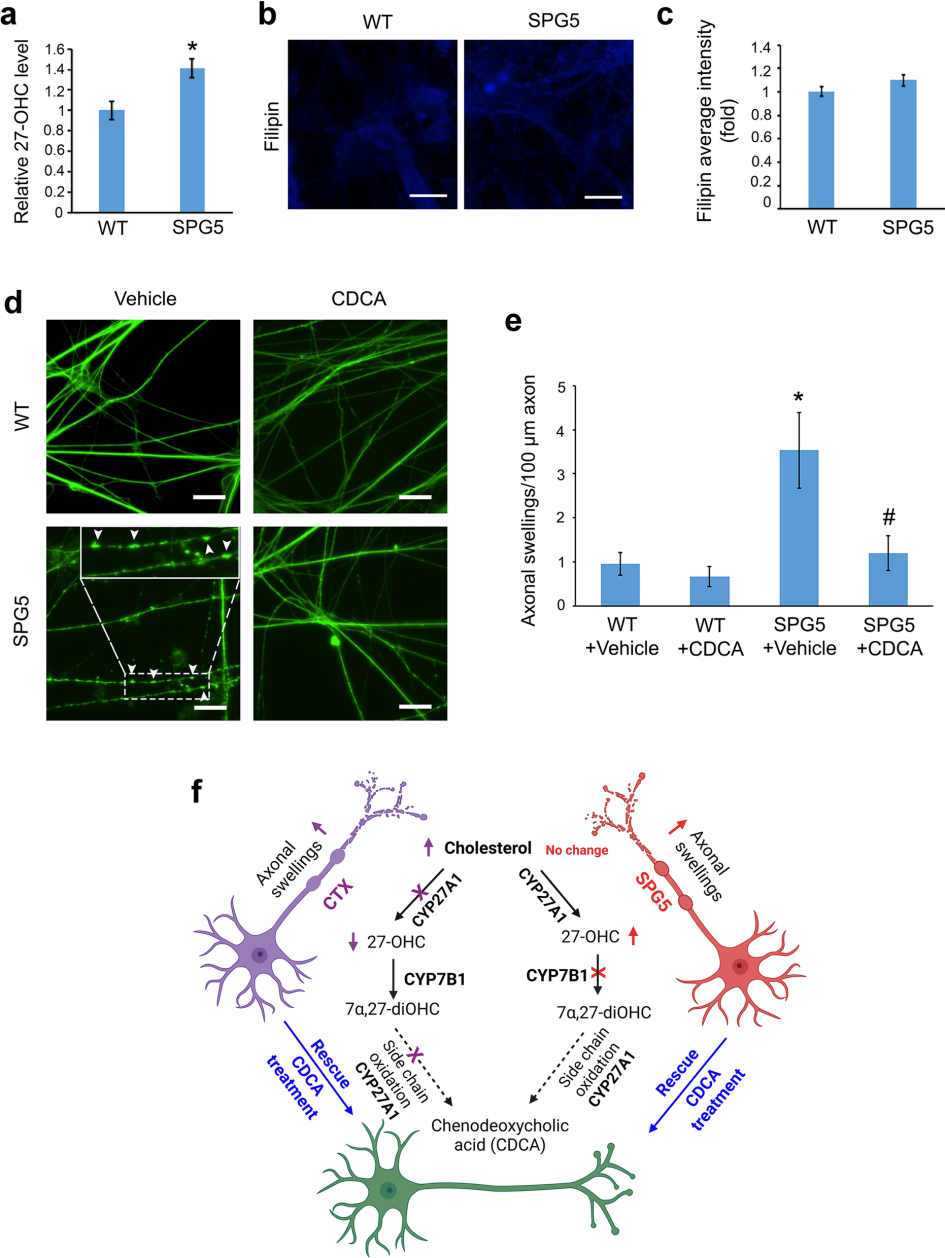
Impaired cholesterol metabolism and axonal swellings in SPG5 cortical PNs. **a** Relative 27-OH-cholesterol levels in SPG5 cortical PNs were significantly increased compared to WT controls. *p < 0.05 compared to WT by two-sided Student *t*-test. **b** Filipin staining was used to determine cholesterol levels in cultures. Representative pictures showed Filipin staining (blue) of WT and SPG5 cortical PNs. Scale bar, 20 µm. **c** Quantification of Filipin average intensity showed similar levels in WT and SPG5 forebrain neurons. **d** Representative pictures of TAU immunostaining (green) in WT and SPG5 cortical PNs after vehicle or CDCA treatment. Axonal swellings are magnified and indicated with arrowheads. Scale bar, 20 µm. **e** Quantification of axonal swellings in WT and SPG5 cortical PNs. Data are represented as means ± SEM. *p < 0.05 compared to WT groups, ^#^*p* < 0.05 compared to SPG5 + vehicle by Tukey’s range test after ANOVA. (**f**) Schematic summary of the iPSC models of SPG5 and CTX (created with BioRender.com)

Cholesterol can be metabolized by CYP7B1 through the ‘acidic pathway’, producing primary bile acids [40]. Initially, cholesterol is oxidized to 27-OHC, and this can be further degraded to chenodeoxycholic acid (CDCA) [41, 42]. Given that 27-OHC is directly downstream of cholesterol metabolism catalyzed by CYP7B1, 27-OHC may be altered in SPG5 iPSCs-derived cortical neurons. Indeed, using the 27-OHC ELISA kit (MyBioSource, Cat. #: MBS285996), we observed a significant increase of 27-OHC levels in SPG5 cortical PNs compared with WT PNs (Fig. 7a). To determine whether total cholesterol levels were affected by the *CYP7B1* mutations, we performed Filipin staining in SPG5 cortical PNs (Fig. 7b, c). Filipin intensity did not show significant alterations in SPG5 cortical neurons as compared with WT neurons, indicating that total cholesterol content was not affected by the *CYP7B1* mutation, in agreement with the known metabolic effects of CYP7B1 (Fig. 7f). Together, our data reveal that SPG5 iPSCs-derived cortical PNs recapitulate disease-specific axonal and biochemical defects, including decreased axonal outgrowth and 27-OHC accumulation, due to the *CYP7B1* mutations.

### Accumulated axonal swellings of SPG5 cortical PNs in long-term culture

Axonal swellings are enlargements within axons, representing the accumulation of transport cargos, and are a common pathogenic hallmark in cellular and animal models of HSP [33–36, 43]. To investigate whether the *CYP7B1* mutations result in axonal swellings in long-term cultured neurons, we performed immunostaining of TAU to visualize axons of 3-month-old SPG5 and WT iPSCs-derived cortical PNs (Fig. 7d). In WT cortical PN cultures, rare axonal swellings were observed. Interestingly, there was a significant increase in the number of axonal swellings in SPG5 neurons as compared to controls (Fig. 7d and e), suggesting that perturbed CYP7B1 activity underlies the accumulated axonal swellings.

Next, we examined whether axonal swellings can be suppressed using the lipid-targeting agent CDCA, particularly given the established effects of CDCA in the clinical treatment of CTX [44–46]. Cells in both control and SPG5 groups attached well to the culture dish after 10 µM CDCA treatment. Importantly, CDCA significantly reduced axonal swellings in SPG5 neurons compared with the vehicle group (Fig. 7d and e). Taken together, patient iPSC-based neuronal model of SPG5, as well as of CTX, exhibited disease-specific defects including accumulated axonal swellings that can be effectively ameliorated by CDCA (Fig. 7f).

### Effects of *CYP7B1-*deficiency on axonal degeneration of human cortical PNs

**Fig. 8:**
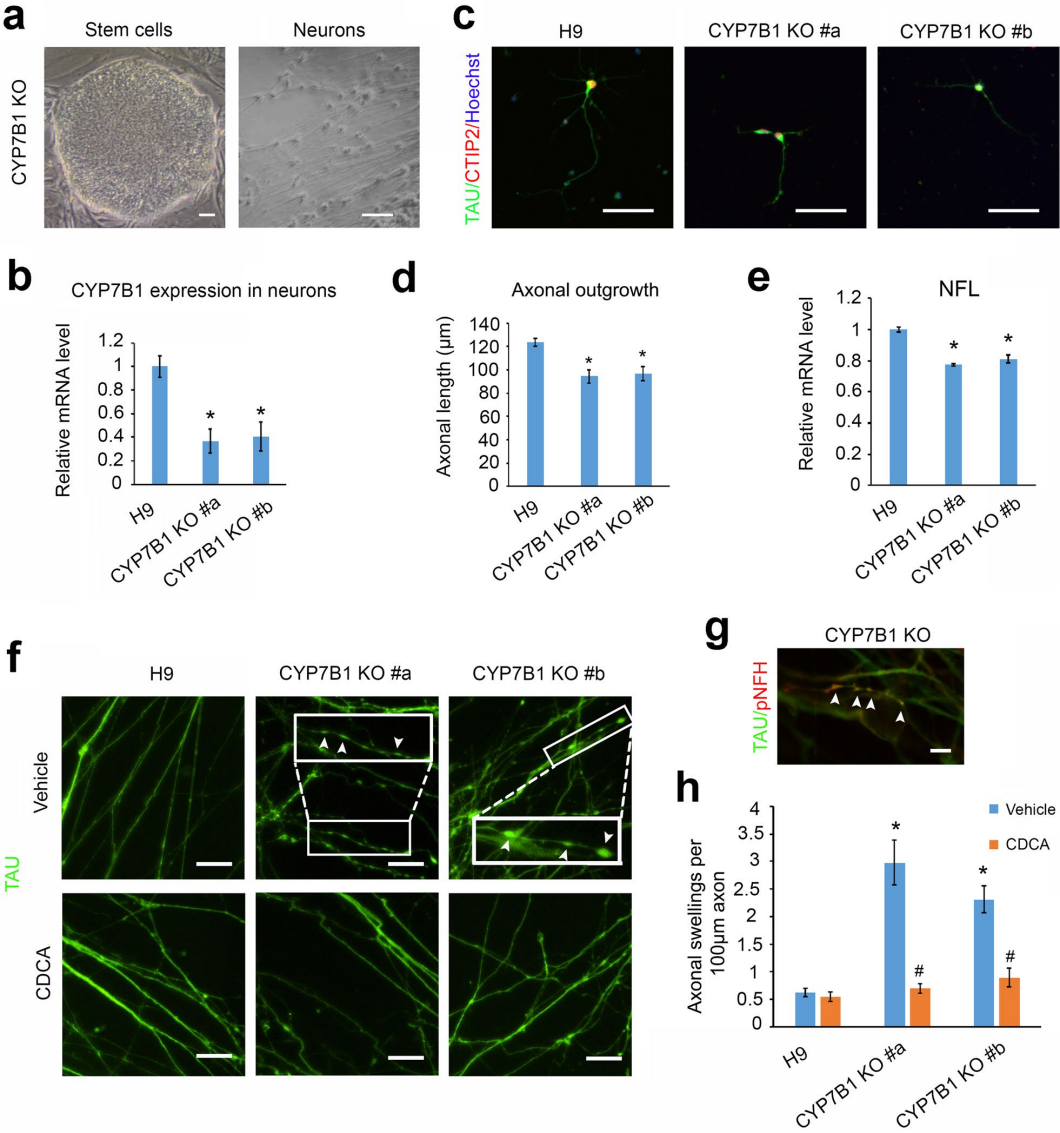
CDCA treatment rescued axonal degeneration induced by loss of CYP7B1. **a** Representative phase-contrast image showing *CYP7B1* KO stem cell clones generated via CRISPR-cas9-mediated gene editing of hESCs. These ESCs were then differentiated into neurons. Scale bar, 50 µm. **b** qPCR showing mRNA expression of CYP7B1 in H9, CYP7B1 KO #a, and CYP7B1 KO #b cortical PNs. **c** Double immunostaining of TAU and CTIP2 in the indicated cortical PNs. Red: CTIP2; green: TAU; blue: Hoechst. Scale bar, 50 µm. **d** Axonal outgrowth quantifications of the indicated cortical PNs. **e** Quantifications of *NFL* expression in the indicated PNs. **f** Representative images showing immunostaining of TAU in H9, CYP7B1 KO #a, and CYP7B1 KO #b cortical PNs after vehicle or CDCA treatment. Axonal swellings are magnified and indicated with arrowheads. Scale bar, 20 µm. **g** A representative image showing double immunostaining of TAU and pNFH in CYP7B1 KO cortical PN axon swellings (arrowheads). Scale bar, 5 µm. **h** Quantification of TAU^+^ axonal swellings in H9, CYP7B1 KO #a, and CYP7B1 KO #b cortical PNs after vehicle or CDCA treatment. Data are represented as means ± SEM. *p < 0.05 compared to H9 neurons treated with vehicle by Dunnett’s test after ANOVA (**b**, **d**, **e,** and **h**). ^#^*p* < 0.05 compared to CYP7B1 KO vehicle-treated group by two-sided Student *t*-test (**h**)

To further determine the role of CYP7B1 in axonal degeneration of human cortical PNs, we established CYP7B1 KO hESC lines using CRISPR-Cas9-mediated gene editing. After drug selection, two CYP7B1 KO clonal lines (CYP7B1 KO #a and #b) were generated and then differentiated into cortical PNs (Fig. 8a). CYP7B1 expression at both mRNA (Fig. 8b) and protein levels (Additional file 1: Fig. S3) was significantly decreased in CYP7B1 KO cortical PNs compared to H9 control neurons, confirming CYP7B1 loss of function in these neurons. Loss of CYP7B1 caused a significant decrease of axonal outgrowth in *CYP7B1* KO neurons (Fig. 8c and d), supporting a role for CYP7B1 in axonal development and extension.

Neurofilament light chain (NfL), the major NF component, is important for normal axonal function. Interestingly, we observed a significant decrease in NfL in 7-week CYP7B1 KO cortical PNs compared with H9 neurons (Fig. 8e), suggesting that loss of CYP7B1 may impair cytoskeleton organization, leading to the degeneration of cortical PNs in SPG5. Indeed, in long-term cultures, there was a significant increase in the number of axonal swellings in CYP7B1 KO neurons as compared to control neurons (Fig. 8f and h). Moreover, TAU^+^ swellings were double stained for pNFH, a marker for disorganized cytoskeleton and degenerated axon (Fig. 8g). Intriguingly, CDCA treatment significantly reduced the axonal swellings in CYP7B1 KO cortical PNs (Fig. 8h). In sum, these data indicate that CYP7B1 loss induced axonal degeneration through, at least partially, altering cytoskeletal organization, and that CDCA can rescue the axonal degeneration in these neurons.

## Discussion

Impaired cholesterol homeostasis in the CNS contributes to neurological disorders such as CTX and SPG5, which are caused by biallelic mutations in the *CYP27A1* and *CYP7B1* genes, respectively [17, 47, 48]. To examine the lipid defects in CTX and SPG5 neurons, we generated iPSC lines from skin fibroblasts of CTX and SPG5 patients. CTX and SPG5 iPSCs were efficiently differentiated into cortical PNs expressing CTIP2, a cortical PN marker. CTX cortical PNs exhibited high levels of cholesterol, while SPG5 cortical PNs showed high levels of 27-OHC, recapitulating disease-specific biochemical changes found in CTX and SPG5 patients, respectively (Fig. 7f). Moreover, CTX and SPG5 cortical PNs exhibited axonal degeneration, with reduced axonal length and increased axonal swellings. Treatment with the bile acid CDCA, a downstream product of cholesterol metabolism, mitigated the observed biochemical disturbances and axonal defects. Notably, knocking out the SPG5 gene *CYP7B1* in hESCs resulted in similar axonal defects and cytoskeleton disorganization in human cortical PNs, which also are mitigated by CDCA treatment. Taken together, these data demonstrate the successful establishment of CTX and SPG5 pluripotent stem cell-based neuronal models and reveal a therapeutic effect for CDCA against axonal degeneration of human cortical neurons in both disease models. Hence, CDCA, which is used to treat CTX, may hold promise as a safe neuroprotective drug for clinical use in SPG5 as well.

In brain, cholesterol synthesis, storage and degradation are exquisitely regulated. CYP7B1 (mutated in SPG5) is one of the key enzymes mediating 27-OHC degradation through the acidic pathway of cholesterol degradation, while CYP27A1 (mutated in CTX) is involved in the degradation of cholesterol to 27-OHC. Thus, iPSCs with CYP7B1 or CYP27A1 biallelic mutations provide ideal models to decipher the role of impaired cholesterol metabolism in neuronal function. In SPG5 patients, mutations in *CYP7B1*, a key enzyme in cholesterol degradation, result in 27-OHC accumulation in neuronal cells and liver cells [9]. Excessive 27-OHC is toxic to cells, induces defects in neuronal morphology, reduces neuronal metabolism and generally impairs normal neuronal function [10, 12, 49]. In this study, starting from CTX and SPG5 patient skin fibroblasts and using iPSC-derived cortical PNs, we identified accumulation of cholesterol and 27-OHC, in iPSC-derived neuronal models of CTX and SPG5, respectively, thus demonstrating disease-specific biochemical changes at the cellular level.

Targeting cholesterol and bile acid metabolism to restore homeostasis offers a compelling strategy to prevent neuronal dysfunction in specific neurodegenerative diseases. Though we only have iPSCs from one patient each for CTX and SPG5, the recapitulation of disease-specific biochemical and axonal degeneration in these iPSC models, as well as in the SPG5-related CYP7B1 KO hESC lines, provides unique opportunities to study the direct effects of these biochemical changes on axonal degeneration. In both SPG5 and CTX, gene mutations affect cholesterol and bile acid metabolism, resulting in CDCA deficiency [50]. CDCA is a natural farnesoid X receptor (FXR) agonist that significantly suppresses cholesterol and bile acid biosynthesis through feedback mechanisms and reduces cholesterol accumulation [51, 52]. In a clinical study, abnormal bile acid profiles including reductions of both total serum bile acids and secondary bile acids, such as ursodeoxycholic and lithocholic acids, were improved by CDCA treatment in SPG5 patients [53]. However, the direct effect of CDCA on axonal degeneration in CTX and SPG5 has not been reported yet. In this study, we found that CDCA abrogated the axonal swellings of CTX and SPG5 iPSC-derived cortical neurons, demonstrating a neuroprotective effect of CDCA against axonal degeneration in both diseases. Moreover, cholesterol levels were also reduced after CDCA treatment, suggesting that CDCA can regulate cholesterol synthesis and thus enhance its protective effects in human neurons. Our results are consistent with the improvement of neurological outcome in previous clinical trials of CTX patients treated with CDCA [46, 54], and provide direct evidence for the protective effects of CDCA on CTX and SPG5 human neurons. Though cortical PNs (deep layer, CTIP2^+^) can be efficiently generated from human pluripotent stem cells (over 70%) [55] and were utilized in this study, the direct differentiation and identification of corticospinal motor neurons from iPSCs remains a challenge in the field. When such a paradigm becomes available, it would be interesting to determine whether corticospinal motor neurons would show even more prominent phenotypes than other cortical projection neurons in CTX and SPG5 models.

The axonal degeneration in SPG5 and CTX iPSCs-derived cortical PNs was concurrent with the accumulation of 27-OHC and cholesterol, respectively, indicating that imbalanced cholesterol and bile acid metabolism results in the aberrant neuronal morphology, while the pathomechanisms remain largely unknown. In this study, we observed axonal varicosities and reduced axonal elongation in CTX and SPG5 iPSC-derived neurons. Increased axonal varicosities and swellings are characteristic pathologies caused by impaired axonal transport and accumulated organelles, which has also been observed in models of common forms of HSP [36, 43, 56]. These findings suggest that impaired vesicular transport along axons as well as perturbed axonal extension can occur downstream of cholesterol and bile acid metabolic pathway defects [13, 57] as a common pathomechanism for axonal degeneration in both CTX and SPG5. Moreover, the reversibility of axonal varicosities that the administration of CDCA had on axonal degeneration of CTX and SPG5 iPSC-derived neurons further confirms this hypothesis, indicating that CDCA may carry a therapeutic potential in SPG5, in accordance with CTX. By knocking out *CYP7B1* in hESCs and examining these ESC-derived cortical PNs, our study further revealed impaired neurofilament expression and organization in CYP7B1-knockout neurons. Using electron microscopy, it has been previously shown that axonal swellings contain disorganized neurofilaments [58, 59]. Though the cause-effect relationship between neurofilament disorganization and accumulated axonal swelling awaits further investigation, our data prefigure perturbed neurofilament organization in SPG5. Furthermore, increased axonal swellings of SPG5 neurons can be suppressed by CDCA treatment, providing a therapeutic approach for rescuing axonal degeneration in SPG5, which in essence is a neurometabolic disease like CTX.

## Conclusion

This study established induced pluripotent stem cell-based models of CTX and SPG5 that recapitulate disease-specific lipid disturbances and axonal degeneration. Notably, CDCA can effectively rescue axonal degeneration in both CTX and SPG5 iPSC-derived neurons, providing direct evidence of CDCA’s effects on human nerve cells. By knocking out the SPG5 gene *CYP7B1* in hESCs, this study also found that impaired neurofilament expression and organization are implicated in axonal deficits that can be mitigated by CDCA, suggesting a new disease mechanism and therapeutic target to rescue axonal degeneration. Given the established beneficial role of CDCA in morbidity and prognosis of patients with CTX when the treatment regimen is commenced as early as possible, early initiation of CDCA therapy may also prove to be of value in slowing down or halting disease progression in SPG5, a currently untreatable and inexorably progressive neurologic disease.

## Supplementary Information

**Additional file 1. Figure S1:** Effect of CDCA on total cholesterol content in control neurons. **Figure S2:** Effects of cholesterol treatment on control human iPSC-derived neurons. **Figure S3:** Reduced protein levels in CYP7B1 knockout cells.

## Data Availability

The data that support the findings of this study are available in this study or from the corresponding author upon reasonable request.

## Abbreviations

CNS: Central nervous system
*CYP27A1*: Cytochrome P450 family 27 subfamily A member 1
*CYP7A1*: Cytochrome P450 family 7 subfamily A member 1
*CYP7B1*: Cytochrome P450 family 7 subfamily B member 1
CDCA: Chenodeoxycholic acid
CTX: Cerebrotendinous xanthomatosis
iPSC: Induced pluripotent stem cell
hESC: Human embryonic stem cell
HSP: Hereditary spastic paraplegia
3β-HCA: 3β-Hydroxy-5-cholestenoic acid
25-OHC: 25-Hydroxycholesterol
27-OHC: 27-Hydroxycholesterol
PNs: Projection neurons
SPG5: Spastic paraplegia type 5
WT: Wild-type
