# Supplementary material for: Chenodeoxycholic acid rescues axonal degeneration in induced pluripotent stem cell-derived neurons from spastic paraplegia type 5 and cerebrotendinous xanthomatosis patients
Source: Orphanet J Rare Dis. 2023 Apr 6;18:72. doi: 10.1186/s13023-023-02666-w (PMC10080795; doi:10.1186/s13023-023-02666-w)
Supplement: Supplementary file 1 — Additional file 1. Figure S1: Effect of CDCA on total cholesterol content in control neurons. Figure S2: Effects of cholesterol treatment on control human iPSC-derived neurons. Figure S3: Reduced protein levels in CYP7B1 knockout cells. [file 13023_2023_2666_MOESM1_ESM.pdf]

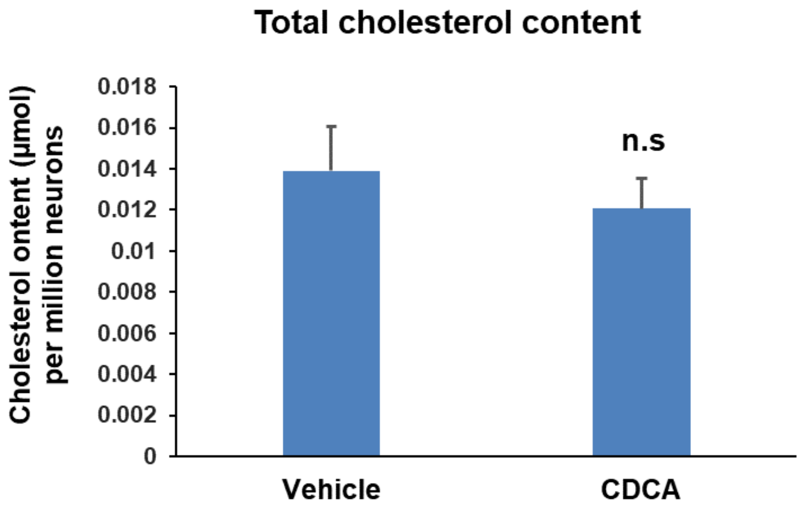

Supplemental Figure 1. Effect of CDCA on total cholesterol content in control neurons. Following treatment with vehicle (DMSO) or CDCA (10 µg/ml) for 1 week, total cholesterol content in WT neurons was compared. There was no statistical difference between these two groups. Data are represented as means ± standard deviation. n.s., not significant.

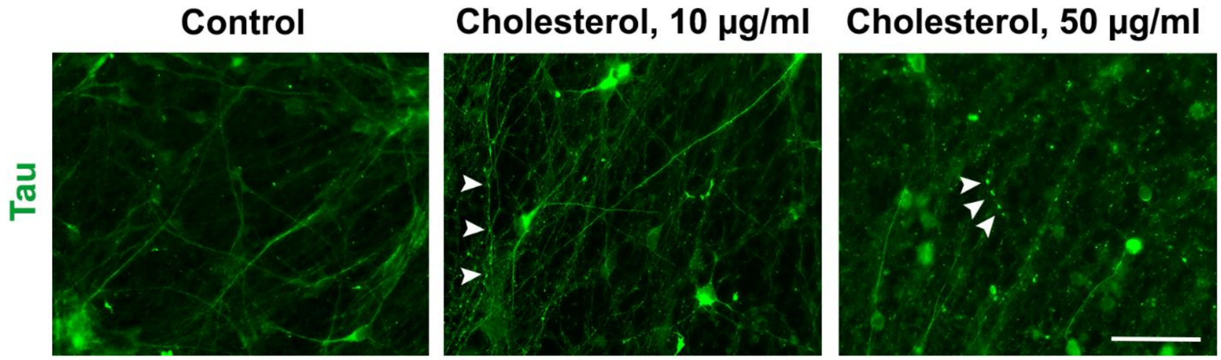

Supplemental Figure 2. Effects of cholesterol treatment on control human iPSC-derived neurons. To examine effects of cholesterol homeostasis on axonal defects, control neurons were treated with two concentrations of cholesterol (10 and 50 µg/ml). After a 2-day treatment, there was dramatic breakdown and retraction of axons in the high dose-treated group. At 3 days after treatment, cells were fixed and stained for TAU. Accumulated axonal swellings (indicated with arrowheads) were observed in both cholesterol-treated groups. Scale bar, 50 µm.

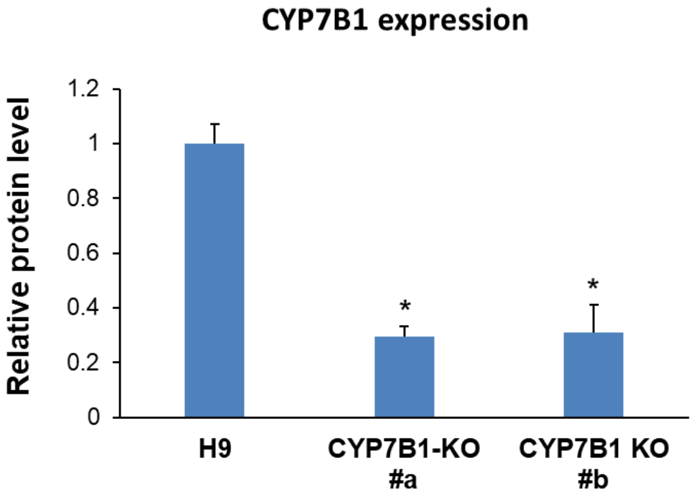

Supplemental Figure 3. Reduced protein levels in CYP7B1 knockout cells. ELISA analysis showing relative expression of CYP7B1 protein in H9 control and CYP7B1 KO cells. There was a significant reduction in the protein levels in both CYP7B1 KO lines as compared to H9 controls. Data are represented as means ± SEM, *$p<0.05$ compared to H9 group by Dunnett's test after ANOVA.
